# Effects of Dandelion Flavonoid Extract on the Accumulation of Flavonoids in Layer Hen Meat, Slaughter Performance and Blood Antioxidant Indicators of Spent Laying Hens

**DOI:** 10.3390/ani15060886

**Published:** 2025-03-20

**Authors:** Yuyu Wei, Jingwen Zhang, Yiming Zhang, Dingkuo Liu, Chunxue You, Wenjuan Zhang, Chaoqi Ren, Xin Zhao, Liu’an Li, Xiaoxue Yu

**Affiliations:** 1 Key Laboratory of Intelligent Breeding, Ministry of Agriculture and Rural Affairs (Ministry-Province Joint Establishment), Tianjin Key Laboratory of Agricultural Animal Breeding and Health Breeding, College of Animal Science and Veterinary Medicine, Tianjin Agricultural University, Tianjin 300392, China; weiyuyu0621@163.com (Y.W.); 17695808072@163.com (J.Z.); zhangyimingzym97@163.com (Y.Z.); youchunxue@163.com (C.Y.); zhangwenjuan@tjau.edu.cn (W.Z.); renchaoqi92@163.com (C.R.); zorazz@126.com (X.Z.); 2 Tianjin Key Laboratory of Biological Feed Additive Enterprise, S&E Burgeoning Biotechnology (Tianjin) Co., Ltd., Tianjin 300383, China; liudingkuo1981@163.com

**Keywords:** dandelion flavonoids content, slaughtering performance, blood biochemical indexes, antioxidant capacity, spent laying hens, functional hen meat

## Abstract

This study aimed to investigate the effects of different supplemental amounts of dandelion flavonoid extracts (DFE) in diets on nutrients in chicken, slaughtering performance, blood biochemical indexes and antioxidant capacity of spent laying hens. A total of 180 560-day-old spent Hy-Line Brown laying hens were randomly divided into five groups. The control group was fed the basal diet, while the experimental groups were supplemented with DFE at levels of 1000, 2000, 4000, and 8000 mg/kg (as T1, T2, T3, and T4 group) in the basal diet, respectively. The variables measured included the content of dandelion flavonoids in layer hen thigh meat and breast meat, slaughter performance, blood biochemical indexes, and antioxidant capacity. Data were subjected to a one-way analysis of variance (one-way ANOVA) to assess the impact of DFE supplementation compared to the control group on study outcomes. The results showed that dietary supplementation with DFE can increase the content of dandelion flavonoids in layer hen meat. The contents of rutin in layer hen breast meat of groups T1, T2, T3, and T4 were 1.37, 4.41, 16.26, and 36.03 ng/g, respectively, and the contents of quercetin was 2.58, 1.36, 4.98, 12.48 ng/g. In layer hen thigh meat of groups T1, T2, T3, and T4, the contents of rutin were 11.48, 15.98, 44.43, 122.32 ng/g, and the contents of quercetin were 9.96, 13.14, 23.15, 38.09 ng/g, respectively. The addition of DFE increased the total phenol content of the feed and highly significantly elevated the total phenol content of layer hen meat (*p* < 0.01), and the total phenol content of chicken meat was strongly and positively correlated with the total phenol content of the feed. DFE supplementation significantly decreased abdominal fat percentage (*p* < 0.05) and increased crude fat content in chicken (*p* < 0.05). The addition of DFE reduced aspartate aminotransferase (AST) and alanine aminotransferase (ALT) activities (*p* < 0.05), decreased triglyceride (TG), total cholesterol (TC), low-density lipoprotein (LDL) cholesterol (LDL-C), glucose (GLU), and malondialdehyde (MDA) contents (*p* < 0.05), and increased the content of albumin (ALB), total antioxidant (T-AOC) capacity and total superoxide dismutase (T-SOD), glutathione peroxidase (GSH-Px) activity (*p* < 0.05). Dietary supplementation of DFE at different concentrations could significantly increase the content of dandelion flavonoids in the muscle of spent laying hens, reduce the abdominal fat rate in hens, effectively reduce blood lipid levels, effectively increase crude fat content in thigh muscle, and enhance the body’s antioxidant capacity and liver function.

## Simple Summary

At present, the development and utilization prospects of spent laying hens are broad. Previous research has rarely focused on improving the meat quality and physiological indicators of spent laying hens by adding special active substances. In this study, different concentrations of dandelion flavonoid extract (DFE) were added to the basal diet to observe the differences in flavonoid content in chicken, slaughter performance, basic nutrients in meat, blood biochemical indicators, and blood antioxidant indicators from spent laying hens. The results showed that adding DFE significantly increased the content of dandelion flavonoids in chicken, reduced abdominal fat percentage, increased the crude fat content of chicken, reduced blood lipid content, and improved the body’s antioxidant capacity and liver function. This study provides a reference for improving the economic benefits of spent laying hens, and developing functional chicken products.

## 1. Introduction

The production performance and egg quality of aged laying hens have significantly decreased [1]. Approximately 80–90 week old laying hens enter the elimination period, and these hens in the market will be eliminated to provide hen meat [2]. In China, more than 2 billion spent laying hens are used for meat every year. Muscle of spent laying hens has a high content of crude protein and polyunsaturated fat and low content of fat and cholesterol, which still has a broad prospect for development and utilization [3]. Functional chicken refers to products obtained through active ingredients or specialized breeding methods, which have specific physiological regulatory functions or health benefits, and can satisfy consumer health, nutrition, and functionality demands [4]. If the slaughtering performance and nutrient content of meat are improved by short-term feeding of spent laying hens, and functional chicken is developed, the meat value of spent laying hens can be effectively increased, and the economic benefits can be enhanced.

Dandelion (Taraxacum officinale) is a perennial herbaceous plant of the family of Asteraceae, which is mainly produced in temperate and subtropical regions of the Northern Hemisphere [5]. It is distributed in northern, northeastern, and southwestern China and has been used as a traditional Chinese herb for a long time in the Chinese pharmacopoeia [6]. Dandelion has many functions, including anti-inflammatory, antioxidant, anticancer, immunomodulatory, hypoglycemic, and antibacterial roles [7]. Dandelion in feed increases egg production, improves shell weight and yolk color in laying hens [8]. Main active components in dandelion are flavonoids, phenolic acids, sterols, and polysaccharides [9]. Among them, the representative active substances are rutin and quercetin. Plant flavonoids have been widely used in livestock and poultry production [10,11]. Currently, dandelion flavonoids have a wide range of applications in food, nutritional supplements, nutraceuticals, cosmetics and pharmaceuticals. For example, it is used to produce candies, teas and energy drinks to clear heat and detoxify the body [12]. Dandelion flavonoids have also been used in health supplements to enhance immunity and improve health and in the development of novel drugs to combat tumors, diabetes, and cardiovascular diseases [13].

Nowadays, functional chicken is more in line with consumers’ demand for health and has broad prospects. However, there have been no reports on determining dandelion flavonoids in chicken and developing functional chicken—rich in flavonoids. In this study, different concentrations of DFE were added to the diet for spent Hy-Line Brown laying hens, and then comparative analysis was conducted on the flavonoid content in layer hen breast and thigh meat, slaughter performance, blood biochemical indicators, and antioxidant capacity. The aim of this study was to provide a reference for scientifically guiding the livestock industry to improve the economic benefits of spent laying hens.

## 2. Materials and Methods

The experimental procedures involving spent laying hens were approved by the Animal Care and Use Committee of Tianjin Agricultural University, Tianjin, China, under permit No. 2023LLSC33.

### 2.1. Animals, Diets, and Experimental Design

180 spent Hy-Line Brown laying hens at 560 days of age with similar physiological conditions, weighing 1.85–2.18 kg, were randomly divided into five groups with three replicates of 12 hens each, caged in three-tier cages (W × D × H = 47.5 × 55 × 37 cm per cage), artificially fed, and watered ad libitum, for 30 days. The groups were designated as C group (control), T1, T2, T3, and T4. C group was fed the basal diet (as detailed in Table S1), and groups T1 to T4 were supplemented with 1000, 2000, 4000, and 8000 mg/kg of DFE (13.8% purity) in the basal diet, respectively. Furthermore, nine spent Hy-Line Brown laying hens of 560 days old were gavaged with rutin at a dose of 300 mg/kg and quercetin at a dose of 250 mg/kg. 

### 2.2. Main Materials and Reagents

DFE (purity of 13.8%, batch number PG230418) was purchased from Weinan Dongjiang Tiancheng Industrial Co., Ltd. (Weinan, China). Rutin reference standard (CAS No. 153-18-4, purity ≥ 98%) and Quercetin reference standard (CAS No. 117-39-5, purity ≥ 98%) were purchased from Solarbio Bio-technology Co., Ltd. (Beijing, China). Purified water was purchased from Wahaha Group Co., Ltd. (Hangzhou, China). Reagents for gavage: quercetin (purity 98%), rutin (purity 95%), were purchased from Xi’an Ruihe Biological Engineering Technology Co., Ltd(Xi’an, China). Phosphoric acid, methanol, ethyl acetate, hexane, ethanol, acetic acid, were all purchased from Tianjin Fengchuan Chemical Reagent Technology Co., Ltd., Tianjin, China. The kits for determinate triglycerides (TG) (Item No. A110-1-1), total cholesterol (TC) (Item No. A111-1-1), low-density lipoprotein cholesterol (LDL-C) (Item No. A113-1-1), high-density lipoprotein cholesterol (HDL-C) (Item No. A112-1-1), glucose (GLU) (Item No. F006-1-1), albumin (ALB) (Item No. A028-2-1), malondialdehyde (MDA) (Item No. A003-1-2), and uric acid (UA) (Item No. C012-2-1), the activities of glutathione (GSH-PX) (Item No. A005-1-2), catalase (CAT) (Item No. A007-1-1), total superoxide dismutase (T-SOD) (Item No. A001-3-2), alanine aminotransferase (ALT) (Item No. C009-2-1), aspartate aminotransferase (AST) (Item No. C010-2-1) activities, and total antioxidant capacity (T-AOC) (Item No. A015-2-1) were purchased from Nanjing Jiancheng Bioengineering Institute (Nanjing, China).

### 2.3. Main Instruments

High-performance liquid chromatography (Agilent-1260) and C18 column (250 mm × 4.6 mm, 5 μm) were purchased from Agilent Technologies Ltd., Santa Clara, CA, USA; ultrasonic cleaner (SB-100D) was purchased from Kunshan Ultrasonic Instrument Co., Ltd., Suzhou, China; an NIR meat quality food analyzer (Series 3000) was purchased from MultiScan, Australia; an enzyme labeling instrument (AMR-100T) was purchased from Hangzhou Allsheng Instrument Co., Ltd., Hangzhou, China; a nitrogen blowing instrument (NEVP-111) was purchased from Organomation, Berlin, MA, USA; and a UV-Vis spectrophotometer (UH5300) was purchased from Hitachi Scientific Instruments (Beijing) Co., Ltd., Beijing, China.

### 2.4. Blood Sampling

On the 10th, 20th, and 30th days of the experiment, nine chickens were randomly selected from each group and underwent wing vein blood collection. Blood samples were collected from the wing vein of chickens at 1, 2, 4, 6, 7, and 24 h post gavage. Blood samples were mixed with sodium heparin in anticoagulation tubes, centrifuged at 1096× *g* for 10 min, and then plasma was obtained and stored at −20 °C.

### 2.5. Determination of the Contents of Rutin and Quercetin in Chicken Muscle and Plasma

The separation was performed on a C18 column (4.6 mm × 250 mm, 5 μm) with the mobile phases of methanol (A) and 0.3% phosphoric acid in water (B) at 30 °C and the detection wavelength of 360 nm with the injection volume of 20 μL. The samples were finally filtered through an organic filtration membrane. For detecting rutin in chicken, the procedures of gradient elution were, 0 min—40% A and 15 min—65% A, with a flow rate of 0.8 mL/min. The sample was mixed with 10 folds the volume of 60% ethanol (0.01% acetic acid), vortexed, sonicated at 60 °C for 1 h, centrifuged for 10 min at 2795× *g*, and then extracted again with 1/2 of the extraction solution. The fat was removed with n-hexane. The samples experienced rotatory evaporation, methanol washing, nitrogen blowing, 1 mL of methanol redissolution, and then centrifugation for 10 min at 4382× *g* to obtain the supernatant. For detecting quercetin in chicken, the mobile phase A:B was 60:40 (*V*:*V*) and the flow rate was 0.5 mL/min. The sample was mixed with tenfold the volume of ethyl acetate, vortexed, sonicated at 60 °C for 1 h, centrifugated for 10 min, blown dry with nitrogen, redissolved with 10 mL of 85% methanol, degreased with saturated n-hexane, and centrifugated for 10 min to obtain the supernatant. And then, the supernatant was blown dry with nitrogen, dissolved in 1 mL of methanol and centrifuged to obtain the supernatant. For detecting rutin and quercetin in plasma, the steps of gradient elution were, 0 min—40% A, 15 min—65% A, 20 min—70% A, with a flow rate of 0.8 mL/min. The sample was mixed with half-fold the volume of acetic acid, vortexed, 60 °C ultrasound for 30 min, then added with tenfold the volume of methanol, vortexed, ultrasound for 1 h, centrifugated for 10 min. The supernatant was obtained and blown dry with nitrogen, and then dissolved with 1 mL of methanol and centrifuged to obtain the supernatant.

### 2.6. Determination of Total Phenols in Feed and Layer Hen Meat

The total phenolic content was determined by the Folin–Ciocalteu method [14]. The phenolic compounds in chicken meat and feed were extracted by methanol ultrasonication for 1 h. An amount of 0.1 mL of aliquot was taken and 0.2 mL of Folin–Ciocalteu reagent was added, followed by the addition of 3 mL of 5% sodium carbonate solution, vortexing and mixing of the reaction mixture, and then the reaction was carried out for 1 h at ambient temperature and protected from light, and then the absorbance was measured by a UV-Vis spectrophotometer at 765 nm. The standard curve was prepared using gallic acid control solution with linear regression equation: y = 0.1063x + 0.008 2, R^2^ = 0.9993.

### 2.7. Slaughter Performance Indicators and Chicken Sampling

Chickens were fasted for 12 h. Eight chickens were randomly selected and slaughtered from each group in accordance with the relevant standard) [15]. Pre-slaughter weight, slaughter weight, semi-netted chamber weight, and fully netted chamber weight were weighed. Thigh, breast, abdominal fat, and each organ and intestine were removed weighed. The slaughter rate, semi-clearance chamber rate, full-clearance chamber rate, pectoral muscle rate, leg muscle rate, and abdominal fat rate were calculated. Thigh and breast muscles were taken and stored at −20 °C.

### 2.8. Nutrient Testing in Muscle Tissue

Chicken was prepared into homogenate. The percentage of moisture and the contents of crude protein and crude fat were analyzed through the meat quality analyzer.

### 2.9. Measurement of Blood Biochemical Indicators and Antioxidant Capacity

Plasma was taken for the determination of biochemical indices and antioxidant capacity. An enzyme marker (AMR-100T, Hangzhou, China) was used to measure various blood biochemical parameters. To determine TG, TC, LDL-C, HDL-C, GLU, ALB, MDA, UA levels and GSH-PX, CAT, T-SOD, ALT, AST, T-AOC activities in spent laying hens, commercial kits from the Nanjing Jiancheng Institute of Bioengineering (Nanjing, China) were used. All measurements were carried out following the manufacturer’s instructions.

### 2.10. Statistical Analysis

Data were analyzed using SPSS 26.0, including one-way ANOVA (one-way analysis of variance) and linear regression analysis. One-way ANOVA was used to test for significance (*p* < 0.05 indicates a significant difference, and *p* < 0.01 indicates a highly significant difference) and multiple comparisons were performed by Duncan’s method. Linear regression analysis was used to build a linear regression model and the explanatory power of the model was assessed by Pearson’s correlation coefficient R, coefficient of determination R^2^ and *p*-value. All results are showed as “mean ± standard deviation (SD)”.

## 3. Results

### 3.1. Content of Rutin and Quercetin in Layer Hen Meat and Plasma

**Figure 1:**
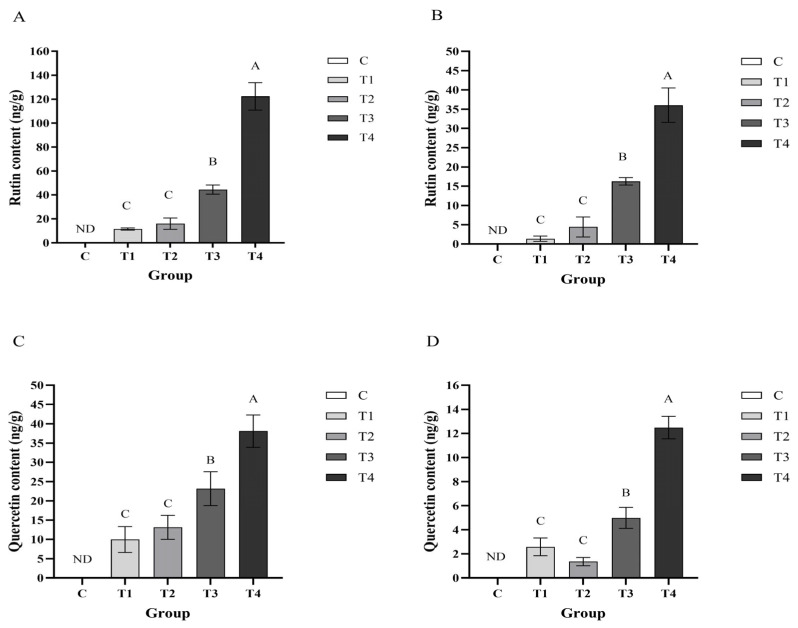
The effect of DFE on the enrichment of rutin in layer hen thigh meat (**A**) and layer hen breast meat (**B**), and the effect of DFE on the enrichment of quercetin in layer hen thigh meat (**C**) and layer hen breast meat (**D**). C, fed with basal diet; T1, supplemented with 1000 mg/kg DFE; T2, supplemented with 2000 mg/kg DFE; T3, supplemented with 4000 mg/kg DFE; T4, supplemented with 8000 mg/kg DFE. A–C indicate extremely significant differences (*p* < 0.01). ND indicates not detected.

As shown in Figure 1, the contents of rutin and quercetin in the breast meat and leg meat of laying hens in group T4 was significantly higher than that in the T1, T2 and T3 groups (*p* < 0.01). The content of rutin and quercetin in the breast meat and leg meat of laying hens in group T3 was significantly higher than that in the T1 and T2 groups (*p* < 0.01). As the DFE supplementation increased, the content of flavonoid in layer hen meat also increased, though it did not show the corresponding multiplicative increase. In layer hen breast meat, the content of rutin in the T1, T2, T3, and T4 groups reached up to 1.37, 4.41, 16.26, and 36.03 ng/g) (Figure 1B), and content of quercetin reached up to 2.58, 1.36, 4.98, and 12.48 ng/g) (Figure 1D), respectively. In layer hen thigh meat, the content of rutin in T1, T2, T3, and T4 groups reached up to 11.48, 15.98, 44.43, 122.32 ng/g) (Figure 1A), and the content of quercetin reached up to 9.96, 13.14, 23.15, 38.09 ng/g) (Figure 1C). As can be seen in Table 1, in the plasma of T4 groups on 10th, 20th, 30th day, the contents of rutin were 26.17, 21.43, and 17.50 ng/mL, and the contents of quercetin were 8.70, 9.19, and 7.83 ng/mL, while the contents of rutin and quercetin in plasma of other groups did not reach above the limit of quantification. Furthermore, in order to investigate the pharmacokinetic pattern of flavonoids in chicken peripheral blood, an oral gavage experiment was carried out. As can be seen in Table 2, rutin in chicken plasma could be detectable at 1 h post gavage, and reached a maximum concentration of 11.94 ng/mL at 4 h post gavage. At 6 and 7 h, the concentration of rutin rapidly decreased, and was not detected at 24 h. Quercetin in the plasma could be detectable at 1 h post gavage, reached a peak of 18.64 ng/mL at 2 h, slowly decreased after 4 h, and was not detected at 24 h post gavage.

### 3.2. Total Phenol Content and Correlation Between Feed and Layer Hen Meat

As shown in Table 3, the total phenol content in the C group feed was 7.05 mg/g, while those in the T1–T4 groups were 7.27, 7.71, 8.27, and 11.46 mg/g, respectively. The total phenol content in feed of the T2, T3, and T4 groups was significantly higher than that of the C group (*p* < 0.01). The total phenol content in layer hen thigh meat was 0.48 mg/g in the control group and increased to 0.60, 1.19, 1.73, and 2.42 mg/g in the T1–T4 groups, respectively. Compared with the C group, T2, T3, and T4 groups showed highly significant increases in thigh phenol content (*p* < 0.01). In layer hen breast meat, the total phenol content was 0.46 mg/g in the C group and rose to 0.51, 0.63, 0.94, and 1.25 mg/g in the T1–T4 groups. The T2, T3, and T4 groups exhibited highly significant increases in breast meat phenol content compared to the C group (*p* < 0.01), while the T1 group showed a significant increase (*p* < 0.05). According to Table 4, the linear regression equations were Y = −2.163 + 0.413X for total phenol content of layer hen thigh meat and Y = −0.700 + 0.175X for total phenol content of layer hen breast meat, with R of 0.892 and 0.890, respectively, indicating strong positive correlations. The R^2^ were 0.795 and 0.792, meaning that feed composition accounted for 79.5% and 79.2% of the variation in thigh and breast meat components, respectively. All regression models were statistically significant (*p* < 0.01). These results demonstrate a high-precision linear response between phenol content in layer hen meat and feed, with the regression models showing good goodness-of-fit and predictive ability, providing a basis for regulating total phenol content in meat.

### 3.3. Slaughtering Performance and Basic Nutritional Composition of Muscle Tissue

As shown in Table 5, compared with that in the C group, the abdominal fat percentage in the T2, T3, and T4 groups significantly decreased (*p* < 0.05). Among these groups, the abdominal fat percentage of the T3 group was the lowest. In the T1 and T4 groups, the crude fat content in thigh muscle significantly increased (*p* < 0.05). Among these groups, the thigh muscle crude fat content of group T1 was the highest. The T1 group had the highest slaughter rate, the T4 group had the highest half-cleaned carcass rate, and the T3 group had the highest full-cleaned carcass rate and thigh muscle rate; however there was no statistical significance (*p* > 0.05). DFE had no significant effect on the crude protein and moisture content of layer hen meat, as well as the crude fat content of breast muscle.

### 3.4. Effects of DFE on Blood Biochemical Indicators

As shown in Table 6, on the 10th day, the content of ALB in the experimental group was significantly higher than that in the C group (*p* < 0.05) with the highest found in the T3 group. On the 20th day, the content of ALB in the T1 group was significantly higher than that in the C group (*p* < 0.05), and the AST activity and the content of LDL-C in the T4 group were significantly lower than those in the C group (*p* < 0.05), and the content of TC in the T1, T2, and T4 groups were significantly lower than that in the C group (*p* < 0.05). On the 30th day, ALT activity in the T3 and T4 groups were significantly lower than that in the C group (*p* < 0.05), ALT activity and the content of TG in the T4 group were significantly lower than those in the C group (*p* < 0.05), and the content of GLU in the T1, T2, and T4 groups was significantly lower than in the C group (*p* < 0.05). The results showed that the dietary supplement of DFE increased the content of ALB, reduced the activities of ALT and AST and the content of LDL-C, TC, TG, and GLU in layer hen plasma, reflecting improved liver function and enhanced lipid metabolism.

### 3.5. Effects of DFE on Antioxidant Capacity

As shown in Table 7, on the 10th day, the contents of MDA in the T2, T3, and T4 groups were significantly lower than that in the C group (*p* < 0.05), with the lowest in the T4 group. The activity of GSH-Px in the test groups was significantly higher than that in the C group (*p* < 0.05), with the highest in the T3 group. On the 20th day, the content of MDA in the T4 group was significantly lower than that in the C group (*p* < 0.05), and the T-AOC capacity of the T3 and T4 groups was significantly higher than that of the C group (*p* < 0.05). On the 30th day, the T-SOD activity in the T3 and T4 groups was significantly higher than that in the C group (*p* < 0.05). These results showed that the addition of DFE reduces the products of peroxidation, increases the activity of antioxidant enzymes, and strengthens the antioxidant function.

## 4. Discussion

Nutritional and functional feed additives are important for improving livestock production. The aim of this study was to investigate the effects of DFE on flavonoid content in layer hen meat, slaughter performance, nutrients in muscle tissue, blood biochemical indices and antioxidant capacity of spent Hy-Line Brown laying hens.

In traditional Chinese medicine theory, aged hens has abundant nutritional and restorative properties, and are considered edible for women in labor, the old and weak, and those who have endured prolonged illness. The development of flavonoid-rich functional layer hen meat can provide higher economic benefits and nutritional values for spent laying hens. In this study, the rutin and quercetin are enriched in layer hen breast and thigh meat of the test groups, while the rutin and quercetin could be detected only in the plasma of the T4 group. Gavage test results showed the rutin and quercetin in the plasma could be detected at 1 h later, and the concentrations increased rapidly and then decreased slowly. Twenty-four hours later, rutin and quercetin could not be detected in the plasma. It can be seen that rutin and quercetin are metabolized quickly in the blood, with low blood concentrations, and are more distributed in muscle tissue. In this study, quercetin peaked at 2 h post gavage and rutin peaked at 4 h post gavage, followed by gradual metabolism, which is consistent with the previous results [16,17]. The results of this test showed that rutin and quercetin could be detected in plasma and muscle of aged hens supplemented with DFE additives. The metabolism of rutin occurs mainly in organs such as the intestine, the liver, and the kidneys [18]. Quercetin is metabolized mainly in the small intestine and liver, the small intestine being the main site of absorption [19], where the glycoside is hydrolyzed and absorbed, and in the liver where quercetin undergoes metabolism such as glucuronidation, with derivatives and unmetabolized compounds being released into the circulation through the hepatic portal vein [20]. Rutin and quercetin have low water solubility, limiting their bioavailability in vivo. However, they can bind to plasma proteins and undergo glucuronidation, allowing them to circulate in peripheral blood. This enables them transport to various tissues and organs to perform biological functions.

In this experiment, a positive correlation was found between the polyphenol content of poultry feed and total phenolic compounds in layer hen meat. This effect may be related to the direct deposition of plant polyphenols in muscle tissue after absorption through the intestine, or to the indirect enhancement of nutrient absorption through modulation of intestinal morphology [21]. Several studies have demonstrated that the addition of plant polyphenols or extracts to the feed can significantly increase the total phenol content of layer hen meat. For example, the addition of marjoram extract to broiler feed resulted in a dose-dependent increase in the total phenol content of breast and leg meat with the amount of the additive, which was consistently higher than that of the control during storage [22]. The addition of a 1.0% gallic and linoleic acid dietary mixture to broiler feed resulted in a significant increase in the total phenol content of breast meat in the experimental group compared to the control [23]. According to Nagendra Prasad [24], there was a remarkably strong relationship between polyphenol content and total antioxidant capacity, with the R^2^ reaching 0.9773. Additionally, previous studies have shown that dietary sources rich in phenolics play a significant role in enhancing the antioxidative properties of meat. Oregano, rosemary, sage essential oils and grape pomace are phenolic substances that show significant antioxidant activity in lamb and broiler meat [25,26]. In addition, polyphenols have significant antioxidant and antimicrobial properties, which can effectively inhibit oxidative reactions and microbial growth in meat, thus prolonging shelf life [27].

Slaughter performance is a measure of growth performance and nutrient deposition efficiency of livestock and poultry. No research has been performed on the potential of DFE to enhance the productivity of broilers or laying hens. The main bioactivities of DFE are antioxidant, lipid-lowering, anti-inflammatory and immune-enhancing effects, which theoretically would improve animal slaughter performance. Muscle and fat are the main components of broiler carcasses, but abdominal fat and subcutaneous fat, which are of lower economic value, are considered to be the main sources of waste in poultry production [28]. In addition, abdominal fat deposition has negative effects on reproductive function, including decreased gamete viability and reduced fertility [29]. It has been shown that leaner hens exhibit better reproductive traits with higher fertility and hatchability rates [30]. In this experiment, the abdominal fat rate of hens in the T2, T3, and T4 groups was significantly lower than that in the C group, suggesting that DFE can reduce abdominal fat in aged hens. However, there had no significant effects on slaughter rate, semi-clearance rate, full-clearance rate, breast muscle rate, and thigh muscle rate in DFE supplemented groups. The content of moisture, crude protein, crude fat, and other components in meat determines its nutritional value. Previous studies have shown that flavonoids have a similar chemical structure to estradiol-17β (E2), and E2 promotes the increase in visceral fat and leptin [31]. Flavonoids can competitively bind to estrogen receptors and exert estrogen-like or anti-estrogenic effects [32]. Zhan et al. [33] showed that high levels of rutin could produce effects similar to E2, stimulating leptin secretion and affecting nutrient absorption and metabolism, thereby increasing the crude fat content of 3-month-old goat meat. Tan et al. [34] reported that dietary supplementation of 2 g/kg dandelion extract in juvenile golden pompano *Trachinotus ovatus* resulted in a significant increase in crude fat content of the whole body of the fish. However, Xu et al. [35] reported that quercetin had no significant effect on the basic nutrient composition of the meat of grass carp. The results of this experiment showed that dietary addition of DFE significantly increased the crude fat content of chicken thigh, which is in agreement with the results of Zhan et al. [33]. The increase in crude fat content in meat helped to enhance the flavor of aged hens, making the taste tender and juicy. However, there was no significant effect on the basic composition of layer hen breast meat, probably because 80-week-old Hy-Line Brown laying hens have a slow metabolism, less efficient absorption and utilization of nutrients; some active ingredients may be very effective in young animals, but their functions may be weakened in aged animals. The improvement effect of feed additives on meat production efficiency and meat quality in aged animals is not as obvious as in the rapid growth stage. In addition, plant-based feed additives, such as sage, oregano, and rosemary, are commonly utilized in livestock and poultry farming to enhance animal health, boost production performance, and elevate meat quality [36]. Rosemary extract is a highly effective antioxidant and has shown significant effects in delaying aldehyde formation, positively affecting the sensory quality of chicken meat [37]. Lopez-Bote found that incorporating 500 mg/kg of rosemary and sage extracts into broiler feed can diminish the extent of lipid oxidation [38]. Reports indicate that adding oregano oil to the diet of broiler chickens can serve as an antibiotic alternative, expedite weight gain, improve the nutritional and biochemical metabolism of chicken meat, and subsequently enhance meat quality [39]. Plant-based feed additives have broad application prospects.

Blood biochemical indexes are important features reflecting the physiological state of the organism. As the key metabolic organ in animals, the liver has an important influence on overall health and production performance. The ALB content reflects the liver’s ability to synthesize proteins to some extent. Low ALB levels usually indicate abnormal liver function [40]. In this study, plasma ALT and AST in the T4 group were significantly lower than those in the C group, and plasma ALB content in the T3 and T4 groups was significantly higher, suggesting that the dietary addition of DFE had a protective effect on the hepatic function of layer hens, probably because DFE prevents the lipid peroxidation of the cell membrane, inhibits the effects of the enzymes AST and ALT into the plasma, and promotes the synthesis of ALB. Previous studies have shown that rutin significantly alleviates cadmium induced chicken liver injury. The ALT and AST activities in the rutin supplemented group were significantly reduced compared to the cadmium poisoned group, indicating that rutin has a direct protective effect on chicken liver function [41]. In addition, after induction with LPS in chicken embryos, quercetin can downregulate the expression level of AMPKα2 protein in the liver, thereby alleviating liver inflammation [42]. The results in this study are consistent with those in previous studies. Flavonoids from vegetables and medicinal plants can lower blood glucose and blood lipids. In this study, serum TG, LDL-C, and GLU contents were reduced, which suggests that the addition of DFE may have hypolipidemic and hypoglycemic abilities. It has been shown that dandelion root and leaf extract has the potential to prevent and relieve obesity-associated NAFLD by significantly reducing hepatic lipid accumulation in mice and can reduce HFD-induced TG, TC, insulin, serum fasting blood glucose levels through activation of the adenosine monophosphate-activated protein kinase (AMPK) pathway [43]. Noor et al. [44] reported that dietary supplementation with 1‰ and 2‰ dandelion leaf powder reduced TG and TC levels in broiler chicks. The reduction in blood glucose and lipid levels in DFE-treated spent laying hens may be related to its inhibition of pancreatic lipase activity to reduce fat absorption [45]. Lipid peroxidation damages cells, destroys cell membranes and organelles, disrupts signaling pathways, and causes metabolic disorders and decreased immunity in the body, which has been associated with the pathogenesis of many diseases [46]. In the present study, T-AOC levels in the T3 and T4 groups were higher, suggesting that DFE plays an important role in preventing lipid oxidation. Superoxide Dismutase (SOD) and GSH-Px are key antioxidant enzymes that protect cells from oxidative stress. SOD catalyzes the disproportionation reaction of superoxide radicals [47], GSH-Px reduces hydrogen peroxide and other lipid peroxides [48]. The results showed that the levels of T-SOD and GSH-PX in the T3 and T4 groups were significantly higher than in the C group, indicating that DFE can enhance the antioxidant capacity by increasing antioxidant enzyme. The above results are consistent with the following study, which showed that the serum MDA, GSH, and SOD levels of dandelion-treated liver-injured mice were normalized [18]. In addition, the antioxidant function of golden pompano *Trachinotus ovatus* fed 1.00 g/kg dandelion extract supplementation was significantly enhanced [21]. The reason dandelion has a significant effect on antioxidant function may be that rutin and quercetin, as flavonoids, can enhance the activity of antioxidant enzymes in various ways. They can directly scavenge free radicals [49] and upregulate the gene expression of antioxidant enzymes by activating the transcription factor NF-E2-related factor 2 (Nrf2) [50]. Moreover, they could inhibit signal pathways like nuclear factor κB (NF-κB) and mitogen-activated protein kinase (MAPK) to decrease the production of reactive oxygen species (ROS) [51,52]. Additionally, rutin and quercetin can enhance the fluidity of cell membranes, strengthen their defense against ROS, and protect antioxidant enzymes from damage [49,53]. Dandelion extracts has been reported having antioxidant and anti-inflammatory effects in lipopolysaccharide (LPS)-stimulated RAW 264.7 cells [54], which significantly reduced NO production without cytotoxicity, and also restored the activities of SOD, CAT, GSH-Px, glutathione Reductase (GSH-GR), and inhibited the expression of iNOS genes and its transcription factor, NF-κB, thereby suppressing oxidative stress and inflammatory responses. These results also confirmed the positive effects of DFE in animal production, reflecting that high levels of DFE can effectively reduce blood lipid levels and enhance the body’s antioxidant capacity and liver function.

## 5. Conclusions

In conclusion, the dietary addition of DFE increased its enrichment in layer hen meat and plasma, with the highest content of rutin and quercetin in layer hen breast and thigh meat and plasma in the T4 group. The dietary addition of DFE increased the total phenolic content in the breast and thigh of layer hens, and there was a linear positive correlation between the total phenolic content in layer hen meat and feed. Dietary addition of DFE reduced abdominal fat percentage and increased crude fat content of thighs in layer hens. DFE decreased plasma TC, TG, LDL-C, MDA content and AST and ALT activities, and increased T-AOC capacity, CAT, T-SOD and GSH-Px activities in layer hens. The changes in these indices demonstrated the effectiveness of DFE in reducing blood lipids and improving antioxidant capacity, liver function in layer hens. These findings support the potential of DFE as an effective feed additive, which provides a practical reference for improving the economic benefits of spent laying hens, the development of functional layer hen meat products with rich dandelion flavonoids.

## Figures and Tables

**Table 1:** Effect of dietary addition of DFE on the content of plasma rutin and quercetin.

	Group	C	T1	T2	T3	T4
Items						
Rutin (ng/mL)	10 d	ND	ND	ND	ND	26.17 ± 2.46
	20 d	ND	ND	ND	ND	21.43 ± 0.68
	30 d	ND	ND	ND	ND	17.50 ± 2.85
Quercetin (ng/mL)	10 d	ND	ND	ND	ND	8.70 ± 2.25
	20 d	ND	ND	ND	ND	9.19 ± 1.87
	30 d	ND	ND	ND	ND	7.83 ± 1.51

C, fed with basal diet; T1, supplemented with 1000 mg/kg DFE; T2, supplemented with 2000 mg/kg DFE; T3, supplemented with 4000 mg/kg DFE; T4, supplemented with 8000 mg/kg DFE. ND indicates not detected.

**Table 2:** Effect of gavage test on the content of plasma rutin and quercetin levels.

	Time	1 h	2 h	4 h	6 h	7 h	24 h	*p*- Value
Items								
Rutin (ng/mL)		3.82 ± 0.08 ^c^	2.80 ± 1.11 ^cd^	11.94 ± 1.96 ^a^	6.21 ± 1.69 ^b^	1.25 ± 0.60 ^c^	ND	<0.01
Quercetin (ng/mL)		5.15 ± 1.97 ^AB^	18.64 ± 7.34 ^A^	17.39 ± 3.67 ^A^	5.67 ± 2.54 ^AB^	5.60 ± 2.87 ^AB^	ND	<0.01

A,B, indicate extremely significant differences between mean values for a given parameter (*p* < 0.01), a–d, different lowercase letters indicate significant differences (*p* < 0.05). ND indicates not detected.

**Table 3:** Effect of dietary addition of DFE on total phenol content of layer hen meat.

Items	C	T1	T2	T3	T4	*p*-Value
Feed (mg/g)	7.05 ± 0.12 ^D^	7.27 ± 0.12 ^D^	7.71 ± 0.13 ^C^	8.27 ± 0.07 ^B^	11.46 ± 0.27 ^A^	<0.01
layer hen thigh meat (mg/g)	0.48 ± 0.12 ^D^	0.6 ± 0.19 ^D^	1.19 ± 0.23 ^C^	1.73 ± 0.23 ^B^	2.42 ± 0.18 ^A^	0.01
layer hen breast meat (mg/g)	0.46 ± 0.07 ^Cd^	0.51 ± 0.08 ^Cc^	0.63 ± 0.10 ^Cc^	0.94 ± 0.17 ^Bb^	1.25 ± 0.13 ^Aa^	0.01

A–C, Different uppercase letters indicate extremely significant differences between mean values for a given parameter (*p* < 0.01); a–d, Different lowercase letters indicate significant differences (*p* < 0.05).

**Table 4:** Correlation between feed and total phenol content of layer hen meat.

Items	Linear Regression Equation	R	R^2^	*p*-Value
layer hen thigh meat	Y = −2.163 + 0.413X	0.892	0.795	<0.01
layer hen breast meat	Y = −0.700 + 0.175X	0.890	0.792	<0.01

Y represents total phenol content in meat and X represents total phenol content in feed. The Pearson correlation coefficients (R) positive, indicating a positive correlation, with a strong correlation range of 0.8 to 1.0; and 0.6–0.8 is strongly correlated; 0.4–0.6 is moderately correlated; 0.2–0.4 is weakly correlated; 0–0.2 is extremely weakly correlated or uncorrelated. R^2^ is used to measure the proportion of variation in the dependent variable that can be explained by the independent variable. R^2^ > 0.6 indicates that the model explains most of the variation and can be used for prediction; R^2^ = 0.3–0.6 indicates that covariates need to be added to enhance the model; R^2^ < 0.2 indicates that although it may be significant, it has low predictive value in practice. *p* < 0.01 indicates that there is a highly significant linear relationship between the independent variable and the dependent variable.

**Table 5:** Effect of dietary addition of DFE on layer hen slaughtering performance and basic nutrient composition of muscle tissue %.

	Group	C	T1	T2	T3	T4	*p*-Value
Items							
Slaughtering rate		79.15 ± 4.56	82.74 ± 2.99	80.62 ± 4.99	81.18 ± 4.48	81.52 ± 1.55	0.48
Half-clearance rate		66.11 ± 2.56	66.05 ± 2.82	66.64 ± 3.58	66.30 ± 4.04	65.16 ± 2.53	0.91
Full bore rate		51.93 ± 1.95	52.41 ± 2.45	53.54 ± 3.19	52.92 ± 3.08	52.42 ± 2.63	0.80
Pectoral muscle rate		16.88 ± 1.73	15.60 ± 2.74	15.76 ± 1.51	16.32 ± 3.27	16.60 ± 3.79	0.87
Thigh muscle rate		24.02 ± 2.25	23.70 ± 1.62	22.98 ± 3.44	24.57 ± 3.09	23.06 ± 3.03	0.76
Abdominal fat percentage		5.49 ± 1.85 ^a^	4.42 ± 2.49 ^ab^	2.89 ± 1.39 ^bc^	1.92 ± 0.52 ^c^	2.99 ± 2.31 ^bc^	<0.01
Thigh muscle coarse protein		20.33 ± 0.39	19.53 ± 0.38	19.80 ± 0.36	19.70 ± 0.45	19.77 ± 0.55	0.09
Thigh muscle moisture		64.90 ± 3.76	61.17 ± 0.9	62.93 ± 2.44	63.33 ± 2.29	63.73 ± 2.11	0.48
Thigh muscle crude fat		8.43 ± 1.75 ^b^	13.23 ± 1.75 ^a^	12.33 ± 2.22 ^ab^	12.45 ± 2.33 ^ab^	12.73 ± 3.41 ^a^	0.20
Breast muscle crude protein		18.53 ± 0.71	18.50 ± 0.35	18.10 ± 0.20	19.13 ± 1.16	18.57 ± 0.50	0.50
Breast muscle moisture		53.77 ± 5.95	56.93 ± 2.78	54.30 ± 2.50	57.60 ± 9.35	56.37 ± 5.01	0.90
Breast muscle crude fat		17.20 ± 2.72	17.47 ± 1.85	19.93 ± 0.70	15.57 ± 3.94	17.07 ± 2.21	0.38

C, fed with basal diet; T1, supplemented with 1000 mg/kg DFE; T2, supplemented with 2000 mg/kg DFE; T3, supplemented with 4000 mg/kg DFE; T4, supplemented with 8000 mg/kg DFE. a–c, Different lowercase letters indicate significant differences between mean values for a given parameter (*p* < 0.05).

**Table 6:** Results of blood biochemistry index power measurements.

	Group	C	T1	T2	T3	T4	*p*-Value
Items and Date							
ALT (U/L)	10 d	13.28 ± 4.71	9.13 ± 2.96	12.93 ± 1.23	12.49 ± 1.05	10.99 ± 6.02	0.44
	20 d	14.08 ± 1.21	12.94 ± 1.63	12.33 ± 3.19	13.64 ± 3.67	13.85 ± 2.21	0.78
	30 d	15.10 ± 2.12 ^a^	14.25 ± 2.23 ^a^	15.04 ± 2.57 ^a^	10.63 ± 1.64 ^b^	10.87 ± 3.75 ^b^	0.02
AST (U/L)	10 d	19.40 ± 5.50	19.38 ± 3.61	19.63 ± 4.59	19.56 ± 5.53	17.98 ± 2.08	0.98
	20 d	23.46 ± 2.39 ^a^	20.71 ± 4.79 ^a^	18.63 ± 5.34 ^ab^	18.21 ± 2.06 ^ab^	14.23 ± 3.79 ^b^	0.03
	30 d	20.18 ± 2.84 ^a^	17.40 ± 2.60 ^a^	17.00 ± 3.68 ^a^	17.31 ± 4.24 ^a^	11.40 ± 5.01 ^b^	0.02
ALB (g/L)	10 d	19.13 ± 1.44 ^b^	23.41 ± 2.27 ^a^	23.94 ± 2.37 ^a^	25.35 ± 2.25 ^a^	23.66 ± 1.10 ^a^	<0.01
	20 d	22.54 ± 1.69 ^b^	25.27 ± 1.89 ^a^	23.76 ± 1.50 ^ab^	22.78 ± 0.77 ^b^	23.33 ± 1.56 ^ab^	0.05
	30 d	23.27 ± 2.72	23.92 ± 3.33	23.43 ± 2.46	22.72 ± 1.66	24.89 ± 2.97	0.67
TG (mmol/L)	10 d	5.07 ± 1.61	4.16 ± 1.41	4.10 ± 1.01	4.18 ± 0.77	3.66 ± 0.53	0.53
	20 d	5.71 ± 0.90	5.40 ± 0.66	4.32 ± 1.41	4.43 ± 0.77	3.99 ± 1.08	0.32
	30 d	5.11 ± 0.77 ^a^	4.73 ± 0.57 ^a^	3.88 ± 0.05 ^ab^	5.04 ± 0.95 ^a^	3.39 ± 0.16 ^b^	0.03
HDL-C (mmol/L)	10 d	1.12 ± 0.69	1.03 ± 0.55	1.31 ± 0.46	1.58 ± 0.96	1.57 ± 0.75	0.63
	20 d	1.31 ± 0.50	1.35 ± 0.57	1.75 ± 1.09	1.81 ± 0.48	2.01 ± 0.29	0.50
	30 d	0.97 ± 0.12	1.04 ± 0.46	1.17 ± 0.51	1.18 ± 0.26	1.11 ± 0.45	0.96
LDL-C (mmol/L)	10 d	1.37 ± 0.34	1.15 ± 0.31	1.27 ± 0.21	1.07 ± 0.38	0.94 ± 0.23	0.18
	20 d	1.55 ± 0.33 ^a^	1.38 ± 0.40 ^a^	1.36 ± 0.54 ^ab^	1.03 ± 0.25 ^ab^	0.83 ± 0.10 ^b^	0.50
	30 d	1.57 ± 0.47	1.29 ± 0.18	1.36 ± 0.71	1.28 ± 0.33	1.19 ± 0.54	0.71
TC (mmol/L)	10 d	2.38 ± 0.56	2.24 ± 0.32	2.16 ± 0.22	2.33 ± 1.19	1.62 ± 0.27	0.26
	20 d	3.31 ± 0.87 ^a^	2.44 ± 0.36 ^b^	2.64 ± 0.53 ^ab^	2.34 ± 0.51 ^b^	2.16 ± 0.59 ^b^	0.03
	30 d	2.27 ± 0.44	2.25 ± 0.44	1.99 ± 0.55	2.06 ± 0.30	1.83 ± 0.43	0.39
GLU (mmol/L)	10 d	13.14 ± 1.94	12.50 ± 2.52	11.42 ± 2.52	12.26 ± 1.36	10.78 ± 1.44	0.30
	20 d	12.73 ± 0.91	12.87 ± 1.31	12.26 ± 0.34	12.01 ± 1.36	11.79 ± 1.39	0.43
	30 d	12.89 ± 1.03 ^a^	10.58 ± 1.62 ^c^	11.06 ± 1.02 ^bc^	12.39 ± 0.93 ^ab^	11.02 ± 1.03 ^bc^	<0.01

C, fed with basal diet; T1, supplemented with 1000 mg/kg DFE; T2, supplemented with 2000 mg/kg DFE; T3, supplemented with 4000 mg/kg DFE; T4, supplemented with 8000 mg/kg DFE. a–c, Different lowercase letters indicate significant differences between mean values for a given parameter (*p* < 0.05).

**Table 7:** Results of antioxidant capacity determination.

	Group	C	T1	T2	T3	T4	*p*-Value
Items							
CAT (U/mL)	10 d	1.48 ± 0.83	1.51 ± 0.93	1.78 ± 0.51	1.78 ± 0.84	1.81 ± 0.56	0.91
	20 d	1.43 ± 0.77	2.13 ± 1.06	2.45 ± 0.65	2.30 ± 0.50	2.46 ± 0.88	0.21
	30 d	1.19 ± 1.01	1.16 ± 0.57	1.19 ± 0.77	1.52 ± 0.33	2.20 ± 0.62	0.15
MDA (nmol/L)	10 d	11.43 ± 2.17 ^a^	10.12 ± 1.46 ^ab^	8.57 ± 3.56 ^b^	5.83 ± 1.53 ^c^	3.69 ± 1.39 ^c^	<0.01
	20 d	11.71 ± 2.18 ^a^	10.83 ± 2.57 ^a^	10.00 ± 1.50 ^a^	8.10 ± 4.16 ^ab^	5.71 ± 3.06 ^b^	0.01
	30 d	11.86 ± 3.48	8.93 ± 1.17	9.64 ± 1.48	10.00 ± 2.02	11.79 ± 2.85	0.15
T-AOC (mmol/L)	10 d	0.29 ± 0.15	0.44 ± 0.20	0.32 ± 0.17	0.33 ± 0.14	0.38 ± 0.08	0.70
	20 d	0.26 ± 0.03 ^b^	0.22 ± 0.10 ^b^	0.24 ± 0.13 ^b^	0.47 ± 0.13 ^a^	0.41 ± 0.12 ^ab^	0.03
	30 d	0.23 ± 0.04	0.29 ± 0.13	0.34 ± 0.07	0.45 ± 0.26	0.39 ± 0.17	0.29
T-SOD (U/L)	10 d	174.11 ± 35.10	183.09 ± 26.25	170.92 ± 7.79	181.7 ± 12.88	209.05 ± 12.97	0.29
	20 d	172.71 ± 25.76	168.52 ± 54.35	184.09 ± 21.10	190.08 ± 17.97	185.49 ± 30.44	0.91
	30 d	164.73 ± 9.41 ^c^	153.34 ± 6.34 ^c^	166.52 ± 6.91 ^c^	203.26 ± 15.87 ^b^	266.36 ± 10.55 ^a^	<0.01
GSH-Px (U/mL)	10 d	2569.55 ± 21.89 ^c^	2860.9 ± 292.71 ^bc^	2985.07 ± 533.11 ^bc^	3792.24 ± 377.01 ^a^	3414.93 ± 240.33 ^ab^	<0.01
	20 d	2388.06 ± 623.96	3094.93 ± 263.03	2712.84 ± 334.60	2789.25 ± 614.01	2779.7 ± 408.04	0.52
	30 d	1977.31 ± 724.82	2459.7 ± 455.89	2880 ± 432.71	2870.45 ± 621.98	2870.45 ± 276.48	0.22

C, fed with basal diet; T1, supplemented with 1000 mg/kg DFE; T2, supplemented with 2000 mg/kg DFE; T3, supplemented with 4000 mg/kg DFE; T4, supplemented with 8000 mg/kg DFE. a–c, Different lowercase letters indicate significant differences between mean values for a given parameter (*p* < 0.05).

## Data Availability

None of the data were deposited in an official repository. Data that support those study findings are available upon request.

## Supplementary Materials

The following supporting information can be downloaded at: https://www.mdpi.com/article/10.3390/ani15060886/s1, Table S1: Composition and nutrient levels of the basal diet (air-dry basis) %.
