# Supplementary material for: Effects of Dandelion Flavonoid Extract on the Accumulation of Flavonoids in Layer Hen Meat, Slaughter Performance and Blood Antioxidant Indicators of Spent Laying Hens
Source: Animals (Basel). 2025 Mar 20;15(6):886. doi: 10.3390/ani15060886 (PMC11939149; doi:10.3390/ani15060886)
Supplement: Supplementary file 1 [file animals-15-00886-s001.zip › animals-3480232-supplementary.pdf]

Table S1. Composition and nutrient levels of the basal diet (air-dry basis) %

Items	Content
Ingredients	
Corn	62.00
Soybean meal	20.35
Peanut meal	4.00
Soybean oil	2.00
Limestone	9.60
$CaHPO_4$	1.15
Met	0.16
NaCl	0.30
Lys	0.16
Trp	0.02
Thr	0.02
Multi-vitamins[1)]	0.04
Mineral elements[2)]	0.20
Total	100.00
Nutrient levels[3)]	
ME/(MJ/kg)	11.75
Crude protein	17.36
Crude fiber	4.20
Crude ash	14.20
Met	0.51
Lys	0.81
Trp	0.20
Thr	0.63
Calcium	3.57
Total phosphorus	0.41

1) The multivitamin content per kg of diet is as follows: VA 13120 IU , VD3 5000 IU , VE 24 IU , VK 2.4 mg , VB1 2 mg , VB2 8.8 mg , VB6 3.2 mg , VB12 0.012 mg , D - Biotin D - Biotin 0. 06 mg , D - Calcium Pantothenate D - Calcium Pantothenate 14 mg , Niacinamide Nicotinic Acid 46.7 mg , Folate 0.8 mg mg , Folic acid 0.8 mg.

2) Minerals were provided as Cu 10 mg , Fe 200 mg , Zn 50 mg , Mn 50 mg per kg of ration.

3) ME and amino acids were calculated values, while the others were measured values.
